# Investigation of sliced body volume (SBV) as respiratory surrogate

**DOI:** 10.1120/jacmp.v14i1.3987

**Published:** 2013-01-07

**Authors:** Jing Cai, Zheng Chang, Jennifer O'Daniel, Sua Yoo, Hong Ge, Christopher Kelsey, Fang‐Fang Yin

**Affiliations:** ^1^ Department of Radiation Oncology Duke University Medical Center Durham NC USA; ^2^ Henan Cancer Hospital Zhengzhou University Henan China

**Keywords:** respiratory surrogate, tumor motion, body volume, 4D CT, motion management

## Abstract

The purpose of this study was to evaluate the sliced body volume (SBV) as a respiratory surrogate by comparing with the real‐time position management (RPM) in phantom and patient cases. Using the SBV surrogate, breathing signals were extracted from unsorted 4D CT images of a motion phantom and 31 cancer patients (17 lung cancers, 14 abdominal cancers) and were compared to those clinically acquired using the RPM system. Correlation coefficient (R), phase difference (D), and absolute phase difference ($D_{A}$) between the SBV‐derived breathing signal and the RPM signal were calculated. 4D CT reconstructed based on the SBV surrogate (4D ${\text{CT}}_{\text{SBV}}$) were compared to those clinically generated based on RPM (4D ${\text{CT}}_{\text{RPM}}$). Image quality of the 4D CT were scored ($S_{\text{SBV}}$ and $S_{\text{RPM}}$, respectively) from 1 to 5 (1 is the best) by experienced evaluators. The comparisons were performed for all patients, and for the lung cancer patients and the abdominal cancer patients separately. RPM box position (P), breathing period (T), amplitude (A), period variability ($V_{T}$), amplitude variability ($V_{A}$), and space‐dependent phase shift (F) were determined and correlated to $S_{\text{SBV}}$. The phantom study showed excellent match between the SBV‐derived breathing signal and the RPM signal (R=0.99, D=−3.0%, $D_{A}=4.5\%$). In the patient study, the mean (± standard deviation (SD)) R, D, $D_{A}$, T, $V_{T}$, A, $V_{A}$, and F were 0.92(±0.05), −3.3% (±7.5%), 11.4% (±4.6%), 3.6 (±  0.8) s, 0.19 (±  0.10), 6.6 (±  2.8) mm, 0.20 (±  0.08), and 0.40 (±  0.18) s, respectively. Significant differences in R and $D_{A}$ (p=0.04 and 0.001, respectively) were found between the lung cancer patients and the abdominal cancer patients. 4D ${\text{CT}}_{\text{RPM}}$ slightly outperformed 4D ${\text{CT}}_{\text{SBV}}$: the mean (±  SD) $S_{\text{RPM}}$ and $S_{\text{SBV}}$ were 2.6 (±  0.6) and 2.9 (±  0.8), respectively, for all patients, 2.5 (±  0.6) and 3.1 (±  0.8), respectively, for the lung cancer patients, and 2.6 (±  0.7) and 2.8 (±  0.9), respectively, for the abdominal cancer patients. The difference between $S_{\text{RPM}}$ and $S_{\text{SBV}}$ was insignificant for the abdominal patients (p=0.59). F correlated moderately with $S_{\text{SBV}}$ (r=0.72). The correlation between SBV‐derived breathing signal and RPM signal varied between patients and was significantly better in the abdomen than in the thorax. Space‐dependent phase shift is a limiting factor of the accuracy of the SBV surrogate.

PACS number: 87.59.bd

## I. INTRODUCTION

Motion management is crucial in radiation therapy (RT) for treating moving tumors.^(^ ^1^ ^)^ Monitoring patient's breathing is a key component of motion management and has been clinically realized via several methods.^(^ ^2^ ^,^ ^3^ ^)^ One example is the real‐time position management (RPM) system (Varian Medical Systems, Palo Alto, CA) which uses reflective markers to track patient's abdomen displacement during the breathing.^(^ ^2^ ^)^ Another example is the Calypso system (Calypso Medical Technologies, Inc., Seattle, WA) in which implanted proprietary Beacon electromagnetic transponders transmit radiofrequency waves to provide information on the position and movement of the tumor.^(^ ^3^ ^)^ Although clinically proven to be effective, these methods are not without limitations. The correlation between RPM markers' motion and tumor motion is unclear.^(^ ^4^ ^)^ Implantation of markers/transponders is invasive and could lead to medical risks, including pneumothorax.^(^ ^5^ ^)^ Furthermore, these methods typically require extra equipment and demand additional staff effort, which increase the cost of treatment.

There is a great interest in seeking alternative methods of monitoring patient's breathing to overcome the above mentioned limitations. Respiratory surrogates based on image features, such as lung air volume, lung air density, deformable image registration (DIR), and normalized cross‐correlation (NCC) have thus been investigated.^(^ ^6^ ^–^ ^11^ ^)^ A universal advantage of the image‐based respiratory surrogates is the elimination of a breathing monitoring device and invasive procedure, since the breathing signal is extracted directly from the images. Using image‐based surrogates for 4D imaging may significantly simplify the simulation process, reduce cost, and improve the efficiency of CT scanner usage.

Since the human body expands and contracts during the breathing cycle, body volume (BV) has been recognized as a potential respiratory surrogate. Compared to lung air volume and lung air density, BV is a more robust surrogate as it can be applied to both thorax and abdomen. Using 3D body surface imaging technique, Hughes et al.^(^ ^12^ ^)^ derived breathing signals based on VisionRT‐Surface‐Derived‐Volume (VRT‐SDV) and found good correlation (r>0.80) between VRT‐SDV and spirometry. Li G. et al.^(^ ^13^ ^)^ examined external torso volume change (TVC) and lung air volume change (AVC) and found that there is a high correlation (r=0.992±0.005, p<0.0001) between the two. In a different study, Li R. and his colleagues^(^ ^14^ ^)^ evaluated four internal respiratory features, including air content, lung density, lung area, and body area (BA), to extract breathing signals for 4D CT sorting. They found that the breathing signal extracted from BA highly correlated with the RPM signal. The mean correlation coefficient between the two was 0.82±0.18, the highest among the four surrogates. It should be noted that in R. Li's study, the BA was calculated as the body area within the body contour in the 2D image slice. It was essentially a 2D counterpart of B V. In the current study, we labeled BA as sliced body volume (SBV) in order to differentiate it from the body surface area. Unlike BV, SBV can be determined directly from the acquired 2D images without using external monitoring devices such as VisionRT. The SBV surrogate thus has the potential advantage of simplicity over the BV surrogate. However, the accuracy and robustness of the SBV surrogate remains incomplete due to limited research on this topic. A systematic investigation of the SBV surrogate is highly desirable. It is the aim of this study to comprehensively evaluate the SBV surrogate by comparing it to a clinical standard (the RPM system), and to examine potential factors that affect the accuracy of the SBV surrogate.

## II. MATERIALS AND METHODS

### A. Extraction of the breathing signal using the SBV surrogate

Evaluation of the SBV surrogate was performed using unsorted 4D CT images of phantom and patients. Since the unsorted 4D CT images represent slice‐by‐slice consecutive acquisitions of human anatomy during the breathing, tracking the changes of SBV provides a continuous trace of the breathing status of the patient. All unsorted 4D CT images were acquired in the cine‐mode on a GE four‐detector CT scanner (LightSpeed Plus 4, GE Healthcare, Waukesha, WI), along with the RPM system and Advantage4D software (GE Healthcare, Milwaukee, WI).

**Figure 1:**
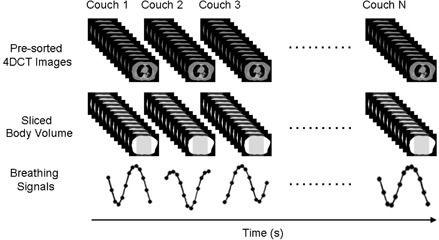
Workflow of the extraction of breathing signal from unsorted 4D CT images using the SBV surrogate. For illustration purpose, only one slice is shown per couch position.

Figure 1 illustrates the workflow of the extraction of the breathing signal. Firstly, body contour was determined for each CT image by applying a threshold and then performing morphological operations to exclude extraneous pixels due to noise. SBV is the total area (or simply, the total number of pixels) encompassed by the body contour. Secondly, an individual breathing curve was generated for each slice by plotting the SBV value as a function of image acquisition time. The means of the SBV values were set to zero. Since our CT scanner images four slices simultaneously, the breathing signal for each couch position was the average of the four breathing curves of the four slices (for illustration purpose, Fig. 1 shows only one slice per couch position). Thirdly, the complete breathing signal was generated by sequentially appending all individual breathing curves. Since there was an approximately 2.3 s couch movement time between consecutive couch positions, the complete breathing signal has gaps between the individual breathing curves.

### B. calculation of respiratory phases from SBV

A low‐pass filter was firstly applied to remove variations induced by cardiac motion and any sudden movements. Respiratory phases were calculated in a systematic approach as described below: (1) peak(s) and valley(s) were detected for each individual breathing curve. Neither peak nor valley can be the first or the last data point; (2) if at least two peaks or valleys were detected in the individual breathing curve, a breathing period was calculated as the difference in time between the two peaks/valleys; (3) an average breathing period was determined for the complete breathing signal, and then an average bin size was calculated assuming 10 bins in one breathing cycle; (4) respiratory phases were calculated for each individual breathing curve depending on the scenario — when there are at least two peaks or valleys, the peaks were set to Phase 50% or the valleys were set to Phase 0% and the phases of other data points were linearly interpolated; in case there is only one peak or one valley, the peak was set to Phase 50% or the valley was set to Phase 0% and the phases of other data points were calculated using the average bin size.

### C. Phantom study

**Figure 2:**
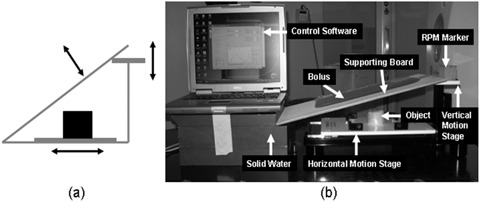
Sketch of the phantom design (a) and a picture of the real phantom and experimental setup (b).

The SBV surrogate was firstly evaluated on an in‐house built motion phantom. The phantom comprises a two‐stage (a horizontal stage and a vertical stage) motion platform (BrainLAB Inc., Feldkirchen, Germany), a cylindrical imaging object placed on the horizontal motion stage, and a 1.0 cm thick bolus piece on a plastic flat board that is propped against the vertical motion stage. Figure 2 shows the sketch of the phantom apparatus and the experimental setup. During the experiment, the motion stages were set to move synchronously in a sinusoid motion pattern. The imaging object moved along with the horizontal stage in the superior–inferior (SI) direction to simulate tumor motion; the bolus piece slid up and down against the vertical stage to simulate body surface movement. The RPM marker box was placed on the vertical stage to acquire the motion signal. The SBV was calculated as the area below the bolus piece and above the horizontal stage. 4D CT images of the phantom were reconstructed based on the SBV‐derived breathing signal (4D ${\text{CT}}_{\text{SBV}}$) and were compared to those reconstructed clinically based on the RPM signal (4D ${\text{CT}}_{\text{RPM}}$). The retrospective sorting algorithm has been extensively described in the literature^(^ ^15^ ^,^ ^16^ ^)^ and will not be repeated here.

### D. Patient study

The SBV surrogate was evaluated in 31 cancer patients (15 male, 16 female, mean age: 67.0) who underwent 4D CT scans with RPM in our institution, among which 17 had lung cancers and 14 had abdominal cancers. For each patient, the breathing signal was extracted from the unsorted 4D CT images using the SBV surrogate and was compared to the RPM signal. Correlation coefficient (R), mean phase difference (D), and mean absolute phase differences ($D_{A}$) between the two were determined. Furthermore, 4D ${\text{CT}}_{\text{SBV}}$ was reconstructed for each patient and compared to the corresponding 4D ${\text{CT}}_{\text{RPM}}$. Image quality of the 4D ${\text{CT}}_{\text{SBV}}$ and 4D ${\text{CT}}_{\text{RPM}}$ was scored (labeled as $S_{\text{SBV}}$ and $S_{\text{RPM}}$, respectively) based on the criteria listed in Table 1 by four evaluators who are experienced radiation oncologists or medical physicists. Score ranges from 1 to 5; 1 is the best. The evaluators were blinded to the surrogate type during their evaluation. All comparisons were performed for the entire group of patients (n=31), and for the lung cancer patients (n=17) and the abdominal cancer patients (n=14) separately. Statistical significance of all comparisons was evaluated using the Wilcoxon signed‐rank test with a significance level of 0.05.

**Table 1:** Criteria for evaluating 4D CT image quality.

*Score*	*Impression*	*Detailed Description*
1	Very Good	Respiratory motion pattern is clear.
		All 10 respiratory phases seem to be sorted correctly.
		Motions of internal organs and structures are very smooth.
		None to minimal image artifacts.
2	Good	Respiratory motion pattern is clear.
		Majority of the respiratory phases seem to be sorted correctly.
		Motions of internal organs and structures are smooth.
		Noticeable image artifacts (do not significantly degrade image quality).
3	Acceptable	Respiratory motion pattern is somewhat clear.
		At least half of the respiratory phases seem to be sorted correctly.
		Motions of internal organs and structures are somewhat smooth.
		Obvious image artifacts (degrade the image quality to some extent).
4	Marginal Acceptable	Respiratory motion pattern is somewhat clear.
		Maybe half of the respiratory phases seem to be sorted correctly.
		Motions of internal organs and structures are not very smooth.
		Strong image artifacts (clearly degrade the image quality).
5	Unacceptable	Respiratory motion pattern is not clear.
		Majority of the respiratory phases seem to be sorted incorrectly.
		Motions of internal organs and structures are not smooth.
		Unacceptable image artifacts (significantly degrade the image quality).

To investigate factors affecting the accuracy of the SBV surrogate, the following parameters were determined from the RPM signal and were correlated to $S_{\text{SBV}}$: RPM box position (P), mean breathing period (T), mean peak‐to‐peak amplitude (A), period variability ($V_{T}$), amplitude variability ($V_{A}$), and space‐dependent phase shift (F). The RPM box position was determined as the level of spinal cord where the RPM box was placed using the anterior–posterior (AP) scout image. For example, if the RPM box was positioned at the level of L1 spinal cord, P was recorded as L1. $V_{T}$ was calculated as the ratio of the standard deviation (SD) of the periods to the mean period. $V_{A}$ was calculated as the ratio of the SD of the peaks and the valleys to the mean peak‐to‐peak amplitude.^(^ ^17^ ^)^ F was estimated as the product of the breathing period and mean absolute phase differences (i.e., $F=T\times D_{A}$). It indicates that different locations of the lungs reach the respiratory peak at different times.

## III. RESULTS

### A. Phantom study

**Figure 3:**
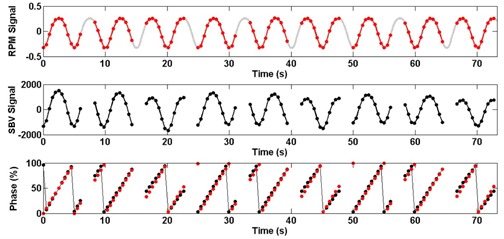
The SBV‐derived motion signal and phases (black) as compared to those determined from the RPM (original in grey, under‐sampled in red) for the phantom study. Gaps in the SBV‐derived motion signal are due to couch movements.

**Figure 4:**
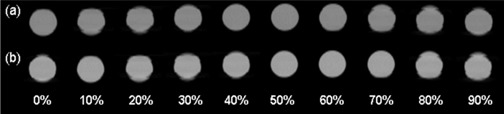
Representative images of 4D ${\text{CT}}_{\text{SBV}}$ (a) and 4D ${\text{CT}}_{\text{RPM}}$ (b) of the imaging object.

The SBV‐derived breathing signal matched well with the RPM signal (R=0.99, D=−0.0%, $D_{A}=4.5\%$, F=0.23 s), as shown in Fig. 3. Image quality of the 4D ${\text{CT}}_{\text{SBV}}$ is comparable to that of the 4D ${\text{CT}}_{\text{RPM}}$ (Fig. 4), with minimal image artifacts shown in both image sets. Motion trajectories of the imaging object determined from the two image sets revealed negligible differences.

### B. Patient study

**Figure 5:**
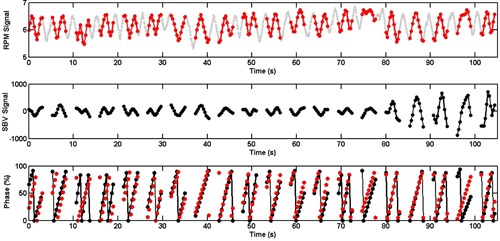
The SBV‐derived breathing signal and respiratory phases (black) as compared to those determined from the RPM (original in grey, under‐sampled in red) for a lung cancer patient. Gaps in the SBV‐derived breathing signal are due to couch movements.

Figure 5 shows the SBV‐derived breathing signal as compared to the RPM signal for a lung cancer patient, revealing that respiratory phases were largely consistent between the two (R=0.92, D=0.8%, $D_{A}=9.4\%$). On average of all 31 patients, the mean (±  SD) R, D, and $D_{A}$ was 0.92 (±  0.05), −3.3%(±7.5%), and 11.4% (±4.6%), respectively. For the lung cancer patients, the mean (±  SD) R, D, and $D_{A}$ was 0.90 (±  0.06),−5.1%(±9.2%), and 13.8% (±4.6%), respectively. For the abdominal cancer patients, the mean (±  SD) R, D, and $D_{A}$ was 0.94 (±  0.03),−1.3%(±4.3%), and 8.5% (±2.6%), respectively. The differences in R and $D_{A}$ were significant (p=0.04 and 0.001, respectively) between the lung cancer patients and the abdominal cancer patients.

**Figure 6:**
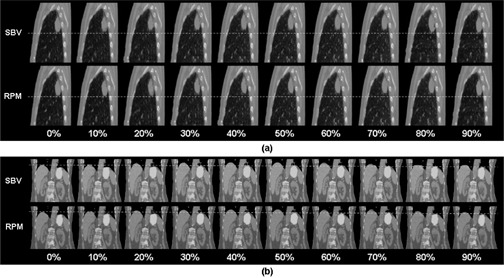
Representative images of 4D ${\text{CT}}_{\text{SBV}}$ and 4D ${\text{CT}}_{\text{RPM}}$ for a lung cancer patient (a) and a liver cancer patient (b). Images are elongated and dash lines are added for better visualization of the respiratory motion.

Figure 6 shows representative images of 4D ${\text{CT}}_{\text{SBV}}$ and 4D ${\text{CT}}_{\text{RPM}}$ for a lung cancer patient (Fig. 6(a)) and a liver cancer patient (Fig. 6(b)). On average, 4D ${\text{CT}}_{\text{RPM}}$ slightly outperformed 4D ${\text{CT}}_{\text{SBV}}$. The mean (±  SD) $S_{\text{RPM}}$ and $S_{\text{SBV}}$ were 2.6 (±  0.6) and 2.9 (±  0.8), respectively, for all 31 patients, 2.5 (±  0.6) and 3.1 (±  0.8), respectively, for the lung cancer patients, and 2.6 (±  0.7) and 2.8 (±  0.9), respectively, for the abdominal cancer patients. The difference between $S_{\text{RPM}}$ and $S_{\text{SBV}}$ was significant for all patients (p=0.04) and for the lung cancer patients (p=0.02), but was insignificant for the abdominal cancer patients (p=0.59).

**Figure 7:**
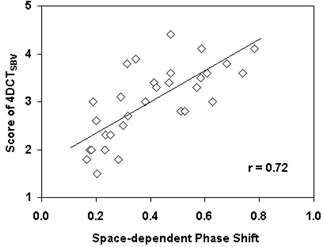
Correlation between 4D ${\text{CT}}_{\text{SBV}}$ image quality ($S_{\text{SBV}}$) and space‐dependent phase shift (F).

P ranged from T12 to S1 and between L1 and L3 for 84% (26/31) of the patients. The mean (±  SD) T, $V_{T}$, A, $V_{A}$, and F was 3.6 (±  0.8) s, 0.19 (±  0.10), 6.6 (±  2.8) mm, 0.20 (±  0.08), and 0.40 (±  0.18) s, respectively. $S_{\text{SBV}}$ correlated moderately with F (r=0.72) (Fig. 7), but not with other parameters.

## IV. DISCUSSION

In this study we comprehensively evaluated the SBV surrogate in a phantom and 31 cancer patients. The phantom study validated our algorithms of signal extraction, respiratory phase calculation, and retrospective sorting. The patient study showed that the correlation between SBV‐derived breathing signal and the RPM signal varied between patients and was better in the abdomen than in the thorax. The SBV surrogate was closely comparable to the RPM in the abdomen, indicating SBV could be used as a single respiratory surrogate for 4D imaging in the abdomen. A potential application of this finding is to use the SBV surrogate in 4D magnetic resonant imaging (4D MRI) for abdominal cancers, which is superior to 4D CT since it is expected to have better soft‐tissue visualization. Using the SBV surrogate for 4D MRI eliminates the requirement for external surrogate devices, reduces the instrument cost, and simplifies the simulation process. Another potential application of the SBV surrogate could be for 4D cone‐beam CT (4D CBCT) imaging at the time of treatment, for which the CBCT projections should yield a reasonable estimate of the SBV. A third application of the SBV surrogate could be to combine with the RPM to improve 4D CT sorting and subsequently its image quality. As the RPM is limited to monitor the breathing only at a specific location, it could misrepresent the breathing status at other locations if the patient breathes irregularly. Conversely, the SBV surrogate provides regional breathing information at different locations. A strategic sorting method that combines the complementary information provided by the RPM and the SBV surrogate can potentially improve the 4D CT image quality. It should be noted that a limitation of using the SBV method compared to the RPM method is that the SBV method cannot be used as a real‐time monitoring system during treatment for either passive monitoring or gating.

Several factors may contribute to the differences between the SBV‐derived breathing signal and the RPM signal. First is the space‐dependent phase shift. This factor does not affect the RPM signal, as the RPM uses a single surrogate to determine the respiratory peaks for all body locations. However, it affects the SBV surrogate since the respiratory peaks were determined separately for different body locations using the images acquired at the corresponding locations. As shown in this study, the image quality of 4D ${\text{CT}}_{\text{SBV}}$ correlated with the space‐dependent phase shift. This finding is contrary to those of previous studies which showed no significant effect between the space‐dependent phase shift and the accuracy of internal respiratory surrogate.^(^ ^11^ ^,^ ^14^ ^)^ This is probably because we studied a larger range of space‐dependent phase shift (0.17–0.78 s) than those previous studies (typically within 0.4 s). The second factor is time‐dependent phase shift (i.e., respiratory peaks may vary from time to time due to patients' breathing variations). This factor could affect the accuracy of both the SBV surrogate and the RPM. However, it is difficult to quantify this parameter and evaluate its effect. In the study, we used the period variability ($V_{T}$) as a close representative of the time‐dependent phase shift and found no correlation between $S_{\text{SBV}}$ and $V_{T}$. In reality, both the space‐dependent and the time‐dependent phase shift may present simultaneously, and the overall effect will be dominated by the space‐dependent phase shift. Another factor is phase calculation error. The accuracy of the phase calculation greatly relies on the satisfaction of the data sufficiency condition (DSC),^(^ ^18^ ^)^ which states that the scan duration at each couch position must be greater than the total time of the average duration of the breathing cycle and the duration of the data acquisition for an image reconstruction. However, the DSC may not be satisfied at all couch positions if the patient has large breathing variations. A fourth factor is the assumption during phase determination that the time from end inspiration to end expiration equals the time from end expiration back to end inspiration. This is not always true in real patients.

The accuracy of SBV surrogate can be improved. In this study, the gaps in the complete breathing signal due to couch movement prohibited analyzing the breathing signal as a whole. The breathing signal had to be analyzed separately per couch position, which was suboptimal and may have caused errors in the phase calculation. It should be noted, however, that this type of error is related to the limitation of the scanner, not to the SBV surrogate itself. If images were acquired with no or smaller gaps, such as in helical‐mode 4D CT scan or in 4D MRI scan, the above‐mentioned problem will no longer exist. In addition, the accuracy of SBV surrogate can be potentially improved by minimizing the patient's breathing variations using audio/video coaching.

## V. CONCLUSIONS

In this study we evaluated the SBV as respiratory surrogate in phantom and 31 cancer patients. Overall, we found that the correlation between the SBV‐derived breathing signal and the RPM signal varied between patients and was significantly better in the abdomen than in the thorax. Space‐dependent phase shift is a limiting factor of the accuracy of the SBV surrogate.
